# Abnormal PTBP1 Expression Sustains the Disease Progression of Multiple Myeloma

**DOI:** 10.1155/2020/4013658

**Published:** 2020-06-18

**Authors:** Hua Bai, Bing Chen

**Affiliations:** Department of Hematology, The Affiliated Drum Tower Hospital of Nanjing University Medical School, Nanjing 210008, China

## Abstract

Multiple myeloma (MM) is a hematopoietic malignancy characterized by heterogeneity, which corresponds to alternative splicing (AS) profiles and disadjust gene expression. Bioinformatics analysis of AS factors possibly related to MM progression identified the polypyrimidine tract binding protein (PTBP1) as candidate. The purpose of this study was to confirm the incidence and prognostic value of PTBP1 in MM patients. Several cohorts of 2971 patients presenting newly diagnosed and relapsed MM were enrolled. Correlations between PTBP1 expression and clinicopathological characteristics, proliferative activity, and response to therapy of myeloma cells were analyzed. Moreover, the effect of PTBP1 on the AS pattern of specific aerobic glycolysis-related genes was explored in MM patients. Clinically, PTBP1 expression was present at all stages; it increased with disease progression and poor prognosis, which was even stronger elevated in patients with high tumor burden and drug resistance. Mechanistically, PTBP1 modulated AS of PKM2 and aerobic glycolysis-related genes in MM patients, which play synergistic or additive effects in clinical outcome. PTBP1 may be a novel marker for prognostic prediction and a promising therapeutic target for the development of anti-MM treatments.

## 1. Introduction

Multiple myeloma (MM) is a plasma cell malignancy and is characterized by hypercalcaemia, renal disorder, anaemia, and lytic bone lesions, and it is incurable. Although the utilities of novel chemotherapies have obviously conferred survival advantage, MM remains a relapsed or refractory disease [1–3]. Thus continued investigations to identify established markers for risk stratification are still in urgent requirement [4]. To date, several molecular markers have been adopted as a standard staging system (DS/ISS); they are still inadequate in prognostic prediction and providing treatment choices [5]. The appropriate biomarkers that can reduce the probability of recurrence and progression are a clinicopathological priority for MM risk stratification [6, 7].

Adaptation to various stresses is an important characteristic of tumor cells. Recent studies reported how tumor cells regulate gene expression at the level of alternative splicing (AS) to withstand various stresses [8, 9]. AS leads to ligation of exons and excision of introns from the pre-mRNA and is arranged by the spliceosome [10]. When the intron/exon boundaries show high standards of conservation, exons are almost contained in the mRNA, whereas some exons lacking consensus sequences are excluded by alternative regulation [11]. In these cases, exons' recognitions are regulated by trans-acting splicing factors (SFs). The main members of SFs are serine-arginine proteins and the heterogeneous nuclear ribonucleoproteins, which act antagonistically in AS regulation [10], and the interaction among antagonistic SFs decides whether exon is skipped or included through AS. Therefore, AS increases the coding potential of genomes and represents an evolutionary advantage [12]. However, the changeable regulation adds further opportunities for error, and the defective splicing may contribute to the neoplastic transformation [13–15].

Polypyrimidine tract-binding protein (PTBP1) is a kind of SFs, which participates in variable biological processes [16]. It has been shown that PTBP1 plays important roles in several tumors, such as bladder cancer [17], pancreatic cancer [18], and colon cancer [19]. Accumulating studies have demonstrated that PTBP1 could modulate the expression of pyruvate kinase M2 isoform (PKM2), which is a vital regulator of glycolysis [16, 19, 20]. For example, Cheng et al. found that PTBP1 knockdown overcomes the resistance to vincristine and oxaliplatin along with the switching of the PKM isoform from PKM2 to PKM1, making for inhibiting glycolysis [19]. However, the role of PTBP1 in MM progression is yet to be elucidated.

In this study, we investigated the impact of PTBP1 expression in MM patients' survival, as well as the correlation with clinicopathological characteristics, proliferative activity, and response to therapy of myeloma cells. We also explored whether PTBP1 plays a functional role in aerobic glycolysis and influences the prognosis in MM.

## 2. Methods and Materials

### 2.1. Data Source and Microarray Analysis

Gene Expression Omnibus (GEO) database was carried out to examine the expression of *PTBP1* in 2971 MM patients (GSE5900 [21], GSE2658 [22], GSE24080 [23], GSE31161 [24], GSE83503 [25], GSE9782 [26], GSE19554 [27], and GSE57317 [28]). Data acquisition and normalization methods in the aforementioned databases have been described previously [23, 29]. The gene expression of *PTBP1* in plasma cells was determined using the Affymetrix U133Plus2.0 microarray, which was performed as previously described [22].

### 2.2. Cell Culture and Western Blotting

Human myeloma cell line (RPMI-8226) was cultured in RPMI 1640 medium (Gibco, USA) supplemented with 10% heat-inactivated FBS (Gibco, USA), penicillin (100 IU/ml), and streptomycin (100 *μ*g/ml) in a humidified incubator at 37°C and 5% CO_2_. Protein extracts and western blotting were performed as described previously [3, 30]. Primary antibodies include PTBP1 (Proteintech, USA), PKM2 (Zen Bioscience, China), and GAPDH (Cell Signaling Technology, USA). GAPDH was used as a loading control to normalize the protein signal. All western blot experiments were repeated in biological triplicate.

### 2.3. Statistical Analysis

Various statistical analysis methods were utilized to assess the roles of *PTBP1* expression in clinical features and prognosis in MM patients. A Kruskal-Wallis test was used to compare multiple sets of samples. A two-tailed Student *t*-test was used to compare the mean values of the two groups. The one-way analysis of variance (ANOVA) test was used to compare means of more than two groups. The chi-square test was used to compare clinical and pathological features between the *PTBP1* high and *PTBP1* low groups. Survival curves were plotted according to the Kaplan-Meier method, and the log-rank test was employed to analyze statistical differences between survival curves. The effect of *PTBP1* expression on outcome was analyzed using univariate and multivariate Cox regression models. For our analyses, the GraphPad Prism 6 software was employed, and *p* ≤ 0.05 was considered statistically significant.

## 3. Results

### 3.1. *PTBP1* Is a High-Risk Myeloma Gene

**Figure 1:**
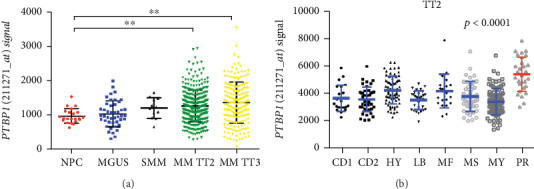
*PTBP1* is a high-risk myeloma gene. (a) *PTBP1* expression of NP (*n* = 22), MGUS (*n* = 44), SMM (*n* = 12), and MM TT2 (*n* = 351) and TT3 (*n* = 208) in GSE5900 and GSE2658. (b) A scatter-plot shows *PTBP1* expression in eight MM subgroups (CD1 and CD2 subgroups with spiked expression of CCND1 and CCND3; PR: proliferation; LB: low-bone disease; HY: hyperdiploid; MS: MMSET; MF: MAFB; MY: myeloid).

To evaluate the potential that PTBP1 is important for MM, we examined the expression of *PTBP1* in normal plasma (NP), smoldering multiple myeloma (SMM), monoclonal gammopathy of undetermined significance (MGUS), and myeloma cells using GEP datasets. Notably, *PTBP1* expression significantly increased from NP, SMM, MGUS, to MM TT2 (Total Therapy 2) and TT3 samples (∗∗*p* < 0.01, Figure 1(a)). In detail, we found higher *PTBP1* expression in the proliferation subgroup (PR); the worst subgroup in MM patients (*p* < 0.0001, Figure 1(b)). These findings led us to confirm that *PTBP1* is a high-risk gene in MM.

### 3.2. Correlations between PTBP1 Expression and Clinicopathological Characteristics

**Figure 2:**
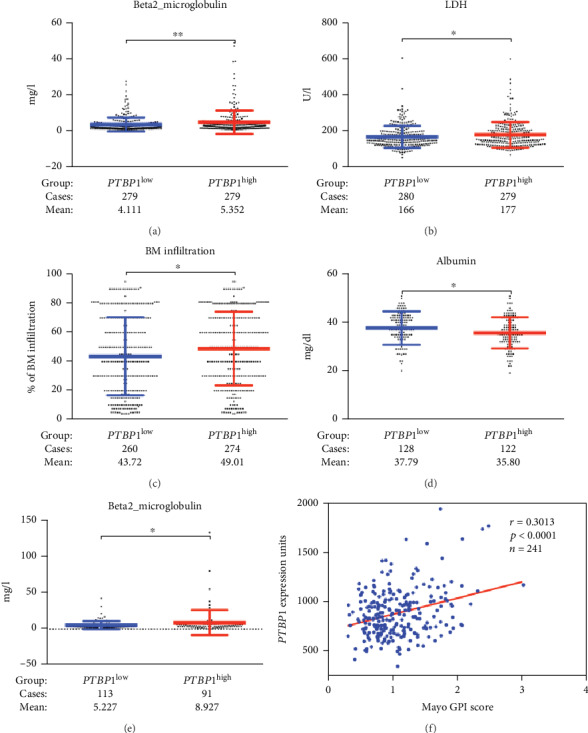
*PTBP1* is linked to myeloma progression in MM. (a)–(c) The levels of *β*2-MG, LDH, and bone marrow infiltration in *PTBP1^high^* and *PTBP1^low^* subgroups. *β*2-MG, LDH, and bone marrow infiltration expressed highest in the *PTBP1^high^* group, while lowest in the *PTBP1^low^* group. (d, e) The levels of ALB and *β*2-MG in the *PTBP1^high^* and *PTBP1^low^* subgroups. ALB expressed highest in the *PTBP1^low^* group, while lowest in the *PTBP1^high^* group. (f) A scatter-plot demonstrating positive correlation of *PTBP1* expression and myeloma proliferation in 246 bortezomib-treated MM patients.

To confirm the robustness of the *PTBP1*, we divided the patients into two categories according to their *PTBP1* expressions (low/high expression, using the 50th percentile as cutoffs) and tested in predicting clinicopathological characteristics distribution. Using 11 clinicopathological characteristics, we found different distributions between the two subgroups in 559 MM patients. Expression levels of *β*2-microglobulin (*β*2-MG), lactate dehydrogenase (LDH), and bone marrow infiltration were significantly increased in the *PTBP1^high^* subgroup compared with the *PTBP1^low^* subgroup by unpaired *t*-test (4.111 ± 0.2308*vs.*5.352 ± 0.3885, *p* = 0.0062, Figure 2(a); 166.5 ± 3.627*vs.*177.5 ± 4.22, *p* = 0.0479, Figure 2(b); and 43.72 ± 1.642*vs.*49.01 ± 1.534, *p* = 0.0188, Figure 2(c)). The remaining characteristics were equally distributed between two subgroups (Table 1). Consistent with GSE24080, *PTBP1* expression was significantly correlated with low albumin (37.79 ± 0.6134*vs.*35.80 ± 0.5878, *p* = 0.0201, Figure 2(d)) and high *β*2-MG levels (5.227 ± 0.5208*vs.*8.927 ± 1.805, *p* = 0.0326, Figure 2(e)) in the GSE9782 (Table 2). To validate our findings in Figure 1(b), we also evaluated the correlation of *PTBP1* expression and proliferation. *PTBP1* expression is positively correlated (*r* = 0.3013, *p* < 0.0001, Figure 2(f)) with myeloma cell proliferation in 246 bortezomib-treated MM patients available at the GSE9782 dataset, using the global gene expression-based proliferation index (GPI) of MM originated by Mayo Clinic as proxy of actual myeloma cell proliferation [31].

### 3.3. Increased PTBP1 Expression Is Linked to Disease Relapse in MM

**Figure 3:**
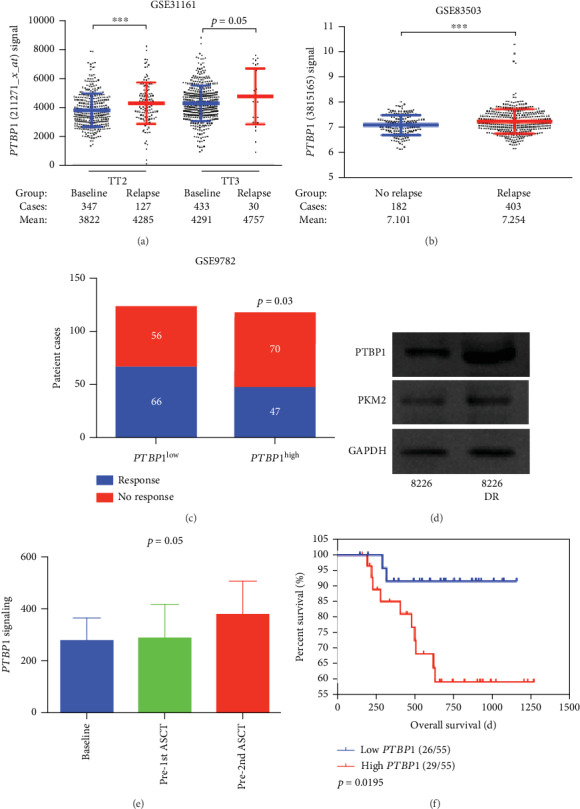
PTBP1 is linked to disease relapse in MM. (a) The expression of *PTBP1* was significantly upregulated in relapsed patients from TT2 and TT3 cohorts in comparison with baseline patients. (b) The expression of *PTBP1* was significantly upregulated in relapsed patients from the GSE83503 cohort in comparison with patients without relapse. (c) Bar view presents response rate between *PTBP1^low^* MM patients and *PTBP1^high^* MM patients treated with bortezomib. (d) Western blots showing the expression of PTBP1, PKM2, and GAPDH in 8226 and 8226-DR cells. (e) The expression of *PTBP1* is showed in 12 MM patient samples collected at diagnosis, pre-1st and pre-2nd ASCT. (f) Kaplan-Meier analyses of OS revealed that high *PTBP1* expression conferred inferior clinical outcomes in TT6 patients.

The expression of *PTBP1* is significantly increased in relapsed MM patients from TT2 and TT3 cohorts compared to baseline patients in the dataset (3822 ± 61.71*vs*. 4285 ± 127, *p* = 0.0003; 4291 ± 59.29*vs*. 4757 ± 350.8, *p* = 0.05, Figure 3(a)).  Figure 3(b) also confirms this and shows the significantly increased *PTBP1* expression in the relapsed group (7.101 ± 0.029*vs*. 7.254 ± 0.023, *p* = 0.0002, Figure 3(b)). Furthermore, Figure 3(c) shows that significantly more patients in the *PTBP1^low^* group had a higher bortezomib-treated response rate (54.1% *vs.* 40.1%, *p* = 0.03). To confirm the correlation between endogenous PTBP1 expression and drug resistance, we used the parental RPMI-8226 (8226) cell line and the RPMI-8226 drug-resistant (8226-DR) cell lines, which acquired drug resistance by prolonged exposure to low doses of bortezomib. As expected, the expression of PTBP1 was substantially upregulated, as determined by western blotting, in 8226-DR cells compared to 8226 cells (Figure 3(d)). Furthermore, message expressions of *PTBP1* in 12 MM patients substantially increased for the following three serials at diagnosis, prior to the first (after chemotherapies) and second autologous stem cell transplant, indicating that increased *PTBP1* may account for drug resistance and promote cell proliferation (*p* = 0.05, Figure 3(e)). Consistent with this finding, the TT6 MM patients, who had been treated with more than one cycle of prior therapy excluding autologous hematopoietic stem cell transplant, were divided into two groups based on high and low *PTBP1* expression, and the high *PTBP1* group had an inferior overall survival (OS) (*p* = 0.0195, Figure 3(f)).

### 3.4. Higher PTBP1 Expression Predicts Poor Prognosis in MM

**Figure 4:**
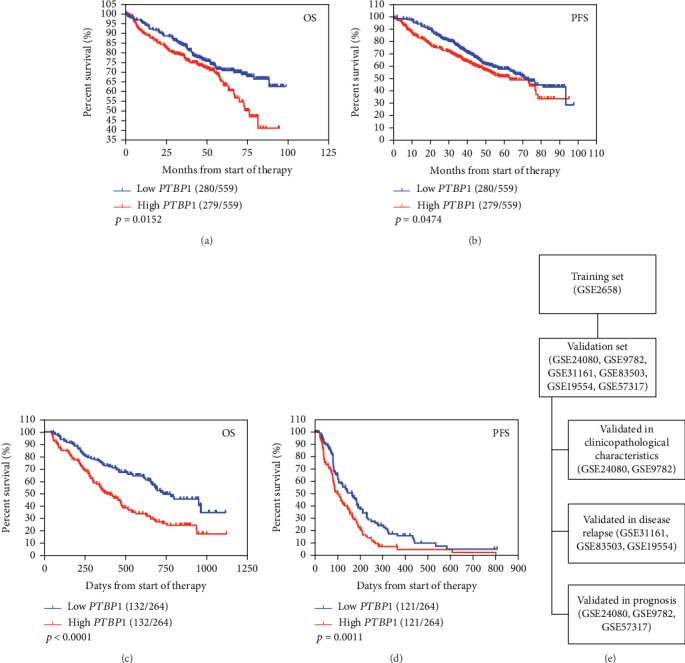
High *PTBP1* expression is linked to a poor prognosis in two independent datasets. (a, b) Kaplan-Meier analyses of OS and PFS revealed that high *PTBP1* expression conferred inferior clinical outcomes in GSE24080. (c, d) Kaplan-Meier analyses of OS and PFS revealed that high *PTBP1* expression conferred inferior clinical outcomes in GSE9782. (e) Bioinformatics flowchart of the GEO database.

To evaluate the biological outcomes of elevated *PTBP1* expression in MM patients, we divided all MM into two groups based on high and low *PTBP1* expression. The high *PTBP1* expression group had shorter median OS and progression-free survival (PFS) time than the low *PTBP1* expression group (44 *vs.* 52 and 39 *vs.* 45, respectively). As shown in Figure 4, MM patients with strong *PTBP1* expression had an inferior OS (*p* = 0.0152, Figure 4(a)) and PFS (*p* = 0.0474, Figure 4(b)). Furthermore, Table 3 shows the impact of *PTBP1* expression and clinicopathological characteristics on clinical outcomes. Based on the results of univariate Cox proportional hazards regression analysis, *β*2-MG, Creatinine (Creat), ALB, and *PTBP1* expression (HR = 1.435, 95% CI: 1.059–1.943, *p* = 0.020) were included in the multivariable Cox proportional hazards regression analysis which indicated that the *PTBP1* expression was still an independent prognostic factor in terms of OS in 559 MM patients (HR = 1.359, 95% CI: 1.001–1.845, *p* = 0.049, Table 3). We also applied the Kaplan-Meier analysis to validated *PTBP1* expression in another independent dataset, and the Kaplan-Meier survival analysis suggested that patients in low *PTBP1* expression group had better OS and PFS compared with those in high *PTBP1* expression group in GSE9782 (*p* < 0.0001, Figure 4(c); *p* = 0.0011, Figure 4(d)).

### 3.5. PKM2 and Other Key Regulators of Warburg Effect Positively Correlate with PTBP1 Expression and Predict Survival in MM

**Figure 5:**
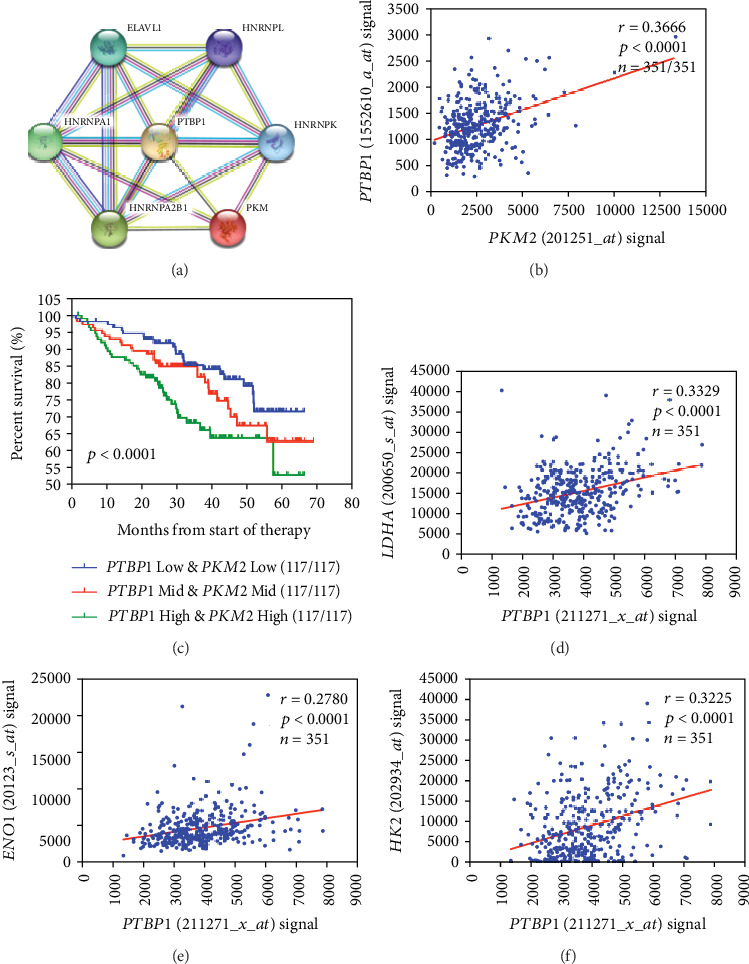
*PTBP1* regulates aerobic glycolysis in MM. (a) The protein network was constructed by online software STRING. (b) A scatter-plot showed the correlation between *PTBP1* and *PKM2*. (c) Kaplan-Meier analyses of OS among MM patients with different expression levels of *PTBP1* and *PKM2*. (d–f) Scatter-plots shows the correlation between *PTBP1* and glycolysis-enhancing genes, respectively.

Using STRING tools, the protein-protein interaction analysis showed that PTBP1 and PKM interact or coexpress in the Homo sapiens protein interaction network (Figure 5(a)). To confirm the correlation between endogenous PTBP1 and PKM2, we investigated the expression of PTBP1 and PKM2 in 8226 and 8226-DR cell lines by western blotting. As expected, both PTBP1 and PKM2 were upregulated in 8226-DR cells compared to 8226 cells (Figure 3(d)). Given that PTBP1 is involved in PKM2 mediated-myeloma progression, we also investigated the correlation between of PTBP1 and PKM2 in MM patients. As shown in Figure 5(b), *PKM2* expression was significantly correlated with *PTBP1* expression in the GSE2658 with *r* value as 0.3666, respectively (*p* < 0.0001, Figure 5(b)). To further investigate whether PTBP1 and PKM2 have synergistic or additive effects in MM patients' outcome, 351 myeloma patients were divided into 3 subgroups including *PTBP1 low/PKM2 low*, *PTBP1 mid/PKM2 mid*, and *PTBP1 high/PKM2 high*, and survival curve showed that the *PTBP1 high/PKM2 high* group has the worst outcome in OS (*p* < 0.0001, Figure 5(c)). These clinical data strongly support findings that *PTBP1* interacts with *PKM2* and promotes its oncogenic function. Previous studies indicated that PKM2 plays a vital role in aerobic glycolysis [32, 33]. We then investigated whether PTBP1 alters aerobic glycolysis by regulating PKM2 expression. The correlation between *PTBP1* and aerobic glycolysis genes was tested in 351 MM patients. The expression of *PTBP1* and glycolysis-enhancing genes, such as lactate dehydrogenase A (*LDHA*), alpha-enolase (*ENO1*), and hexokinase 2 (*HK2*), was significantly positively correlated each other (*r* = 0.3329, *r* = 0.2780, *r* = 0.3225, *p* < 0.0001, Figures 5(d)–5(f)).

## 4. Discussion

MM remains incurable despite novel treatments, and plenty of prognostic markers that reflect tumor- or host-related factors have failed to explain thoroughly the heterogeneity in clinical outcomes [34]. Meanwhile, the AS signature of MM is emerging as a detailed marker to distinguish tumor subtypes and accurately stratify patients [35]. The workflow chart is shown in Figure 4(e); we extracted 2971 MM patients' gene expression microarrays from the GEO database. In GSE24080, we analyzed the association between *PTBP1* and clinicopathological characteristics of 559 MM patients. In GSE24080, MM patients were treated through TT2 (induction therapy: D(T)-PACE, dexamethasone with or without thalidomide; maintenance: thalidomide) and TT3 (induction therapy: VTD-PACE; maintenance: bortezomib-thalidomide-dexamethasone). In GSE2658, we analyzed the expression of *PTBP1* in eight different molecular subgroups. In GSE9782, 264 samples from 264 patients, we analyzed the association between the expression of *PTBP1*, clinicopathological characteristics, and the global gene expression-based proliferation index. In GSE31161, we analyzed the association between *PTBP1* expression and relapse in 937 samples. In GSE83503, 585 samples from 585 cases, we analyzed the expression of *PTBP1* in relapse MM patients and nonrelapse MM patients. In GSE19554, we analyzed the expression of *PTBP1* before and after the first/second transplant in 12 paired MM patients. In GSE57317, 55 samples from 55 cases, we analyzed the association between the expression of *PTBP1* and survival in ASCT-treated MM patients. Therefore, our study showed that PTBP1 favors splicing of oncogenic variants and uncovers novel potential prognostic and therapeutic targets for MM patients.

To predict the prognosis of MM patients, the assessment of cellular proliferative activity is regarded with importance [36]. Proliferation status of MM cells had been evaluated by the plasma cell labeling index, Ki-67, or metaphase cytogenetics [37, 38]. More importantly, *PTBP1* was significantly higher expressed in the PR subgroup, which is characterized by overexpression of proliferation-related genes and accelerate cell cycles, which was the worst prognosis in comparison to the other molecular subgroups [22, 39]. The degree of bone marrow infiltration by MM cells, as estimated by bone marrow biopsy, is one of the considerable determinants of the MM tumor burden [40]. We performed correlation analysis between PTBP1 expression and bone marrow plasma cell infiltration, as derived from bone marrow biopsies. This significant correlation between bone marrow infiltration and PTBP1 expression raises the possibility that PTBP1 may represent a biomarker that indirectly reflects tumor mass and the level of bone marrow invasion at diagnosis [41]. Similarly, we evaluated *BZW2* message levels in 241 bortezomib-treated patients paralleled the myeloma proliferation score, which was scored with the assistance of GPI model constructed by Hose and his associates [42]. Consequently, PTBP1 can facilitate cell proliferation and influences the prognostic impact on MM patients.

Another interesting finding in our study is that *PTBP1* expressions appear to correlate in response to bortezomib-based chemotherapy. Bortezomib, which targets the 26S proteasome subunit *β*5, has induced a high level of positive response rates [43, 44]. However, toxicities associated with global proteasomal inhibition and drug resistance in MM were major concerns, prompting the further development of novel target and therapies. In GSE31161, we found a significant increase in the expression of *PTBP1* in relapsed MM patients from TT2 and TT3 cohorts in comparison with baseline patients. Furthermore, compared to *PTBP1^high^* samples, patients with *PTBP1^low^* MM cells were significantly responded to bortezomib evidenced in GSE9782 [26]. Consistent with GEPs derived from patient populations, the protein expression of PTBP1 was substantially increased in 8226-DR cells compared with parental 8226 cells. The above data suggested that myeloma with higher PTBP1 expression represents more aggressive behavior and worse response to chemotherapies.

Aberrant splicing regulation confers alternative advantage to tumor cells by favoring oncogenic splice variants of tumor-related genes [9, 45]. For example, upregulation of PTBP1 in tumor cells affected glycolytic metabolism by promoting AS of the PKM2 variant [46], leading to acquisition of drug resistance to chemotherapy [18]. Likewise, by screening PTBP1-interaction targets reported by STRING database, we found that *PTBP1* and *PKM2* mRNA expression is positively correlated in GSE2658. Additionally, the high expression of the *PTBP1* and *PKM2* groups showed worst prognosis in various types of MM. The above clinical data forcefully support our findings that *PTBP1* upregulates *PKM2* expression and promotes its oncogenic function. Because PKM2 is a fundamental enzyme for regulation of aerobic glycolysis in tumor cells, we further determine that *PTBP1* expression is positively correlated with aerobic glycolysis genes including LDHA, HK2, and ENO1. Myeloma cells possess increased glycolysis for ATP generation, which is called the Warburg effect [47, 48]. Recently, accelerating studies confirmed that aerobic glycolysis is the hallmark of tumor cells and crucial for proliferation and survival [49, 50]. Despite therapeutic advances, the MM patients eventually relapse and the altered metabolism with increased glycolysis is showed to contribute to drug resistance [48, 51], and increasing research reveals that inhibition of glycolysis restores sensitivity to bortezomib and suppresses tumor growth induced by metabolism [51]. It indicated that targeting glycolysis may be a novel therapeutic strategy to overcome drug resistance.

## 5. Conclusions

In conclusion, our results revealed that increased PTBP1 expression was associated with a poor outcome and resistance to chemotherapy in newly diagnosed MM patients. We also characterized PTBP1 as a novel regulator of aerobic glycolysis which contributes to PKM pre-mRNA splicing. Hence, to better individualize the chemotherapy regime, apart from the laboratory markers of prognostic significance, the incorporation of an initial valuation of PTBP1 expression to an individual prognostic profile for MM risk stratification should be considered.

## Figures and Tables

**Table 1:** Relation of the characteristics in GSE24080.

Characteristic	No. of patients	*PTBP1^low^*	*PTBP1^high^*	*p* value
Age ≥ 65 yr	136/559 (24)	59/280 (21)	77/279 (27)	0.072†
Male sex	337/559 (60)	178/280 (63)	159/279 (56)	0.111†
*β*2 − MG ≥ 3.5 (mg/l)	239/559 (42)	104/280 (37)	135/279 (48)	0.007†
CRP ≥ 4 (mg/l)	292/559 (52)	137/280 (48)	155/279 (55)	0.127∗
Creat ≥ 1.2 (mg/dl)	182/559 (32)	82/280 (29)	100/279 (34)	0.104∗
LDH ≥ 170 (U/l)	231/559 (41)	104/280 (37)	127/279 (45)	0.048∗
ALB ≥ 3.5 (g/dl)	482/559 (86)	246/280 (87)	236/279 (84)	0.272∗
HB ≥ 11 (g/dl)	312/559 (55)	163/280 (58)	149/279 (53)	0.268∗
ASPC ≥ 40%	283/559 (50)	128/280 (45)	155/279 (55)	0.022∗
BMPC ≥ 50%	269/559 (48)	122/280 (43)	147/279 (52)	0.034∗
MRI ≥ 3 lesions	305/559 (54)	149/280 (53)	156/279 (55)	0.552∗

^∗^Fisher's exact test was used. ^†^The chi-square test was used.

**Table 2:** Relation of the characteristics in GSE9782.

Characteristic	No. of patients	*PTBP1^low^*	*PTBP1^high^*	*p* value
Age ≥ 65 yr	87/264 (32)	45/132 (10)	42/132 (31)	0.694†
Male sex	159/264 (60)	76/132 (57)	83/132 (62)	0.378†
Ig A	54/264 (20)	28/132 (21)	26/132 (19)	0.760†
ALB ≥ 40 (mg/dl)	92/264 (34)	57/132 (43)	35/132 (26)	0.004∗
*β*2 − MG ≥ 3.5 (mg/l)	116/264 (43)	56/132 (42)	60/132 (45)	0.619†
CRP ≥ 1.2 (mg/l)	82/264 (31)	37/132 (28)	45/132 (34)	0.287∗

∗Fisher's exact test was used. ^†^The chi-square test was used.

**Table 3:** Univariate and multivariate Cox regression analyses for OS in 559 MM patients.

Variables	Univariate model			Multivariate model		
	HR	95% CI	*p*	HR	95% CI	*p*
Age ≥ 65 yr	1.206	0.885-1.700	0.286			
Male sex	0.968	0.714-1.313	0.835			
*β*2 − MG ≥ 3.5 (mg/l)	2.185	1.613-2.958	0.000	1.870	1.327-2.636	0.000
Creat ≥ 1.2 (mg/dl)	1.731	1.278-2.345	0.000	1.213	0.865-1.701	0.262
ALB ≥ 3.5 (g/d)	0.521	0.360-0.756	0.001	0.640	0.437-0.935	0.021
*PTBP1 ^high^*	1.435	1.059-1.943	0.020	1.359	1.001-1.845	0.049

## Data Availability

The data used to support the findings of this study are available from the corresponding author upon request.

## References

[1] Palumbo A., Anderson K. (2011). Multiple myeloma. *The New England Journal of Medicine*.

[2] Kyle R. A., Rajkumar S. V. (2004). Multiple myeloma. *The New England Journal of Medicine*.

[3] Bai H., Zhu H., Yan Q. (2018). TRPV2-induced Ca2+-calcineurin-NFAT signaling regulates differentiation of osteoclast in multiple myeloma. *Cell Communication and Signaling: CCS*.

[4] Kapoor P., Rajkumar S. V. (2011). Update on risk stratification and treatment of newly diagnosed multiple myeloma. *International Journal of Hematology*.

[5] Greipp P. R., Miguel J. S., Durie B. G. M. (2005). International staging system for multiple myeloma. *Journal of Clinical Oncology*.

[6] Kuiper R., van Duin M., van Vliet M. H. (2015). Prediction of high- and low-risk multiple myeloma based on gene expression and the International Staging System. *Blood*.

[7] Roh J., Shin S. J., Lee A. N. (2017). RGS1 expression is associated with poor prognosis in multiple myeloma. *Journal of Clinical Pathology*.

[8] David C. J., Manley J. L. (2010). Alternative pre-mRNA splicing regulation in cancer: pathways and programs unhinged. *Genes & Development*.

[9] Zhang J., Manley J. L. (2013). Misregulation of pre-mRNA alternative splicing in cancer. *Cancer Discovery*.

[10] Matera A. G., Wang Z. (2014). A day in the life of the spliceosome. *Nature Reviews. Molecular Cell Biology*.

[11] Wang Y., Ma M., Xiao X., Wang Z. (2012). Intronic splicing enhancers, cognate splicing factors and context-dependent regulation rules. *Nature Structural & Molecular Biology*.

[12] Barbosa-Morais N. L., Irimia M., Pan Q. (2012). The evolutionary landscape of alternative splicing in vertebrate species. *Science*.

[13] Cooper T. A., Wan L., Dreyfuss G. (2009). RNA and disease. *Cell*.

[14] Venables J. P., Klinck R., Koh C. (2009). Cancer-associated regulation of alternative splicing. *Nature Structural & Molecular Biology*.

[15] Lee S. C.-W., Abdel-Wahab O. (2016). Therapeutic targeting of splicing in cancer. *Nature Medicine*.

[16] Chen M., Zhang J., Manley J. L. (2010). Turning on a fuel switch of cancer: hnRNP proteins regulate alternative splicing of pyruvate kinase mRNA. *Cancer Research*.

[17] Bielli P., Panzeri V., Lattanzio R. (2018). The splicing factor PTBP1 promotes expression of oncogenic splice variants and predicts poor prognosis in patients with non-muscle-invasive bladder cancer. *Clinical Cancer Research*.

[18] Calabretta S., Bielli P., Passacantilli I. (2016). Modulation of PKM alternative splicing by PTBP1 promotes gemcitabine resistance in pancreatic cancer cells. *Oncogene*.

[19] Cheng C., Xie Z., Li Y., Wang J., Qin C., Zhang Y. (2018). PTBP1 knockdown overcomes the resistance to vincristine and oxaliplatin in drug-resistant colon cancer cells through regulation of glycolysis. *Biomedicine & Pharmacotherapy*.

[20] Hwang S. R., Murga-Zamalloa C., Brown N. (2017). Pyrimidine tract-binding protein 1 mediates pyruvate kinase M2-dependent phosphorylation of signal transducer and activator of transcription 3 and oncogenesis in anaplastic large cell lymphoma. *Laboratory Investigation*.

[21] Zhan F., Barlogie B., Arzoumanian V. (2006). Gene-expression signature of benign monoclonal gammopathy evident in multiple myeloma is linked to good prognosis. *Blood*.

[22] Zhan F., Huang Y., Colla S. (2006). The molecular classification of multiple myeloma. *Blood*.

[23] MAQC Consortium (2010). The MicroArray Quality Control (MAQC)-II study of common practices for the development and validation of microarray-based predictive models. *Nature Biotechnology*.

[24] Mitchell J. S., Li N., Weinhold N. (2016). Genome-wide association study identifies multiple susceptibility loci for multiple myeloma. *Nature Communications*.

[25] Miannay B., Minvielle S., Roux O. (2017). Logic programming reveals alteration of key transcription factors in multiple myeloma. *Scientific Reports*.

[26] Mulligan G., Mitsiades C., Bryant B. (2006). Gene expression profiling and correlation with outcome in clinical trials of the proteasome inhibitor bortezomib. *Blood*.

[27] Zhou W., Yang Y., Xia J. (2013). NEK2 induces drug resistance mainly through activation of efflux drug pumps and is associated with poor prognosis in myeloma and other cancers. *Cancer Cell*.

[28] Heuck C. J., Qu P., van Rhee F. (2014). Five gene probes carry most of the discriminatory power of the 70-gene risk model in multiple myeloma. *Leukemia*.

[29] Bai W., Wang H., Bai H. (2019). Identification of candidate genes and therapeutic agents for light chain amyloidosis based on bioinformatics approach. *Pharmacogenomics and Personalized Medicine*.

[30] Bai H., Chen B. (2020). BAG3 regulates multiple myeloma cell proliferation through FOXM1/Rb/E2F axis. *Cancer Gene Therapy*.

[31] Bergsagel P. L., Kuehl W. M., Zhan F., Sawyer J., Barlogie B., Shaughnessy J. (2005). Cyclin D dysregulation: an early and unifying pathogenic event in multiple myeloma. *Blood*.

[32] Liu F., Ma F., Wang Y. (2017). PKM2 methylation by CARM1 activates aerobic glycolysis to promote tumorigenesis. *Nature Cell Biology*.

[33] Xie M., Yu Y., Kang R. (2016). PKM2-dependent glycolysis promotes NLRP3 and AIM2 inflammasome activation. *Nature Communications*.

[34] Chng W. J., Dispenzieri A., Chim C.-S. (2014). IMWG consensus on risk stratification in multiple myeloma. *Leukemia*.

[35] Trincado J. L., Sebestyen E., Pages A., Eyras E. (2016). The prognostic potential of alternative transcript isoforms across human tumors. *Genome Medicine*.

[36] Isoda A., Kaira K., Iwashina M. (2014). Expression of L-type amino acid transporter 1 (LAT1) as a prognostic and therapeutic indicator in multiple myeloma. *Cancer Science*.

[37] Alexandrakis M. G., Passam F. H., Kyriakou D. S., Dambaki K., Niniraki M., Stathopoulos E. (2004). Ki-67 proliferation index: correlation with prognostic parameters and outcome in multiple myeloma. *American Journal of Clinical Oncology*.

[38] Lokhorst H. M., Boom S. E., Bast B. J. E. G., Ballieux R. E. (1986). Determination of the plasma cell labelling index with bromodeoxyuridine in a double fluorescence technique. *British Journal of Haematology*.

[39] van Andel H., Kocemba K. A., de Haan-Kramer A. (2017). Loss of CYLD expression unleashes Wnt signaling in multiple myeloma and is associated with aggressive disease. *Oncogene*.

[40] Nishida Y., Kimura S., Mizobe H. (2017). Automatic digital quantification of bone marrow myeloma volume in appendicular skeletons - clinical implications and prognostic significance. *Scientific Reports*.

[41] Beldi-Ferchiou A., Skouri N., Ali C. B. (2017). Abnormal repression of SHP-1, SHP-2 and SOCS-1 transcription sustains the activation of the JAK/STAT3 pathway and the progression of the disease in multiple myeloma. *PLoS One*.

[42] Hose D., Reme T., Hielscher T. (2010). Proliferation is a central independent prognostic factor and target for personalized and risk-adapted treatment in multiple myeloma. *Haematologica*.

[43] Oerlemans R., Franke N. E., Assaraf Y. G. (2008). Molecular basis of bortezomib resistance: proteasome subunit *β*5 (PSMB5) gene mutation and overexpression of PSMB5 protein. *Blood*.

[44] Franke N. E., Niewerth D., Assaraf Y. G. (2012). Impaired bortezomib binding to mutant *β*5 subunit of the proteasome is the underlying basis for bortezomib resistance in leukemia cells. *Leukemia*.

[45] Paronetto M. P., Passacantilli I., Sette C. (2016). Alternative splicing and cell survival: from tissue homeostasis to disease. *Cell Death and Differentiation*.

[46] David C. J., Chen M., Assanah M., Canoll P., Manley J. L. (2010). HnRNP proteins controlled by c-Myc deregulate pyruvate kinase mRNA splicing in cancer. *Nature*.

[47] Kato Y., Maeda T., Suzuki A., Baba Y. (2018). Cancer metabolism: new insights into classic characteristics. *Japanese Dental Science Review*.

[48] Panchabhai S., Schlam I., Sebastian S., Fonseca R. (2017). PKM2 and other key regulators of Warburg effect positively correlate with CD147 (EMMPRIN) gene expression and predict survival in multiple myeloma. *Leukemia*.

[49] Koppenol W. H., Bounds P. L., Dang C. V. (2011). Otto Warburg's contributions to current concepts of cancer metabolism. *Nature Reviews. Cancer*.

[50] Vander Heiden M. G., Cantley L. C., Thompson C. B. (2009). Understanding the Warburg effect: the metabolic requirements of cell proliferation. *Science*.

[51] Maiso P., Huynh D., Moschetta M. (2015). Metabolic signature identifies novel targets for drug resistance in multiple myeloma. *Cancer Research*.

